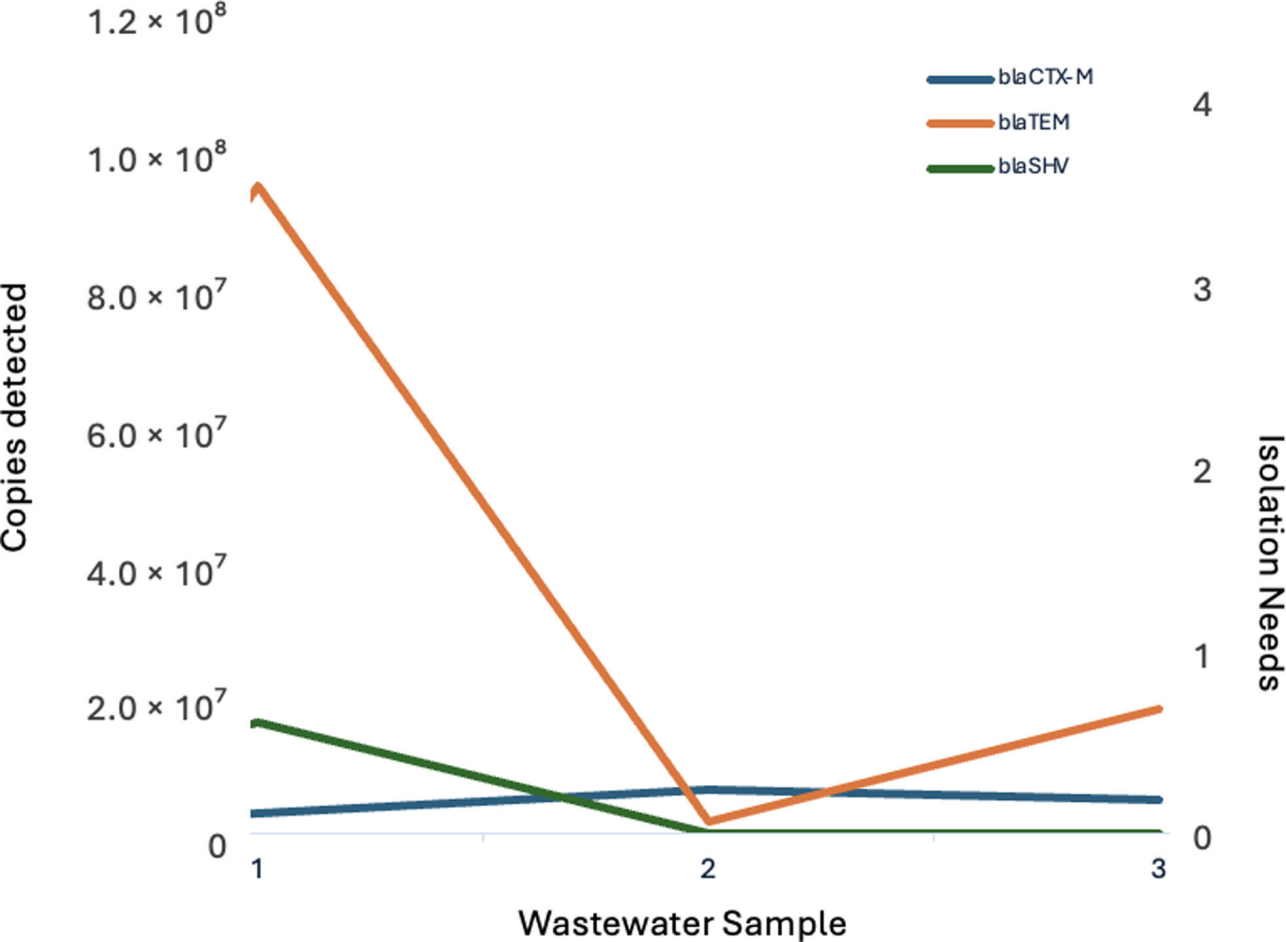# 302 Antibiotic treatment and culture responsiveness for urinary tract infections differ by outpatient clinical setting

**DOI:** 10.1017/ash.2026.10657

**Published:** 2026-06-23

**Authors:** Alfredo Mena Lora, Scott Borgetti, Lior Cohen Yatziv, Mirza Ali, Susan Bleasdale Casey

**Affiliations:** 1 University of Illinois at Chicago; 2 University of Illinios; 3 University of Illinois Chicago; 4 Infection Control Team; 5 N/A

## Abstract

**Background:** Wastewater surveillance has emerged as a powerful tool for monitoring community pathogen burden. However, its role in hospital infection prevention remains unclear. We evaluated hospital wastewater for detectable multidrug-resistant organisms (MDRO) at an academic tertiary-care center (AH) and a community hospital (CH). **Methods:** From April-September 2024, wastewater was collected longitudinally from an AH and a CH. Samples were collected from selective plumbing draining intensive care beds at both facilities. After a 24-hour collection, samples were processed using digital droplet PCR to detect and quantify resistance genes and pathogens, including blaCTX-M, blaTEM, blaCMY-2, blaSHV, IMP, VIM, OXA-48, KPC, NDM, and Candida auris. Results were reported as gene copies per swab. Concurrent hospital isolation logs were reviewed to identify unit isolation with corresponding MDROs at the time of sampling. **Results:** Wastewater from both hospitals consistently contained multiple MDRO genetic markers across all sampling periods (Figures 1-4). Extended-spectrum beta-lactamase (ESBL) genes blaCTX-M, blaTEM, and blaSHV were detected in every sample, often at high copy number. Carbapenemase (CRE) genes IMP, KPC, and VIM were frequently detected, whereas NDM and OXA-48 were present at low and variable levels. C. auris was rarely detected. Across both sites, quantitative gene copy numbers varied over time. AH demonstrated persistently high ESBL and CRE gene burdens with temporal surges (Figure 1-2), while CH showed lower and more episodic detection patterns (Figure 3-4). Isolation needs for ESBL and CRE at both hospitals are reported in Figures 1-4. **Conclusion:** Hospital wastewater harbors diverse MDRO genetic signatures that do not reliably track with isolation practices, reflecting contributions from both silent patient shedding and environmental sources. ESBL genes often remained high even when no patients required isolation, whereas carbapenemase surges frequently preceded increases in CRE isolation, suggesting wastewater may provide early warning of rising resistance pressure. Differences between hospitals likely reflect both patient populations and environmental persistence. Wastewater surveillance may complement infection prevention efforts but should not be interpreted as a direct surrogate for patient colonization. Figure 1. Temporal trends in ESBL-producing resistance genes in wastewater and isolation needs at an academic center Figure 2. Temporal trends in CRE-producing resistance genes in wastewater and isolation needs at an academic center Figure 3. Temporal trends in ESBL-producing resistance genes in wastewater and isolation needs at a community hospital Figure 4. Temporal trends in CRE-producing resistance genes in wastewater and isolation needs at a community hospital

**Figure:**
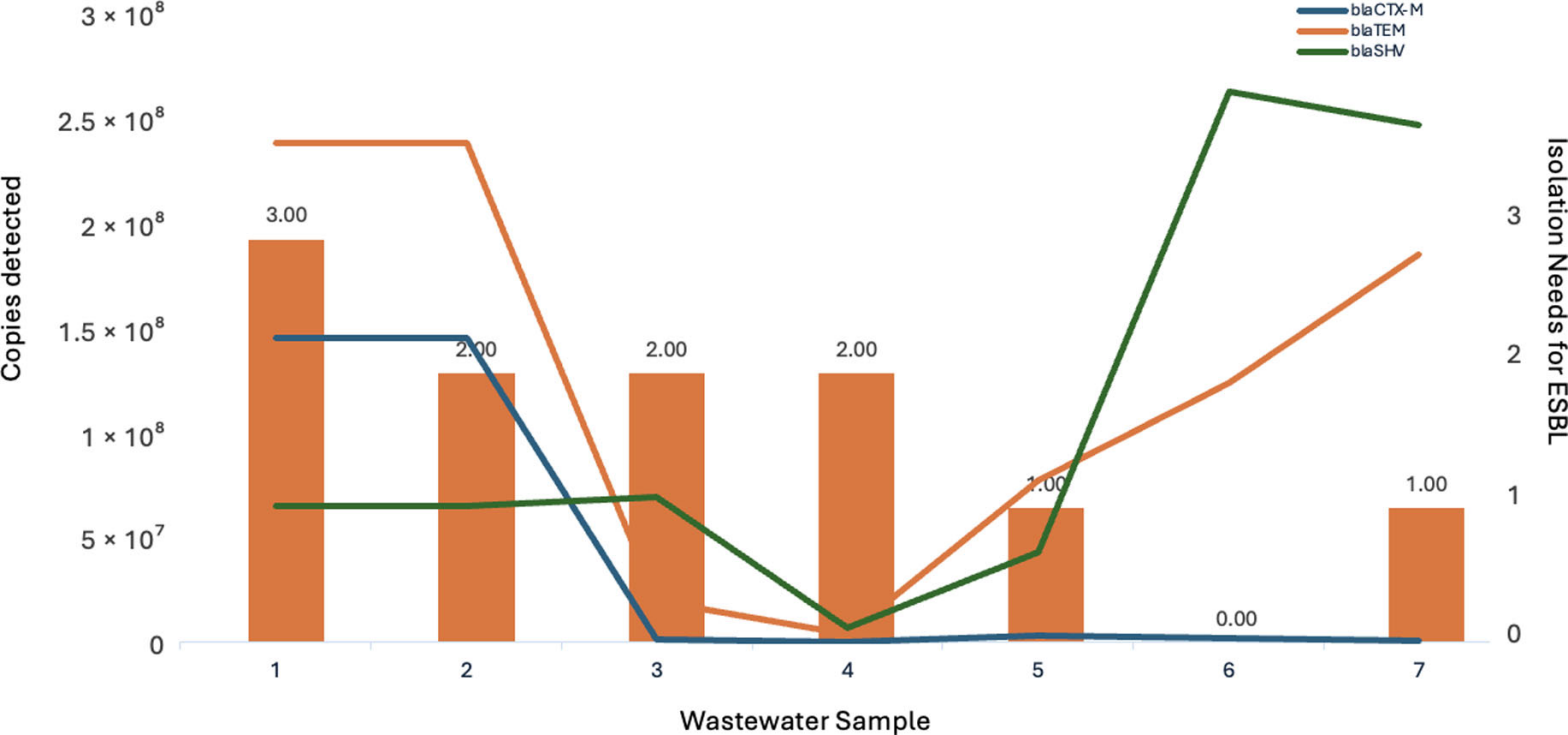

**Figure:**
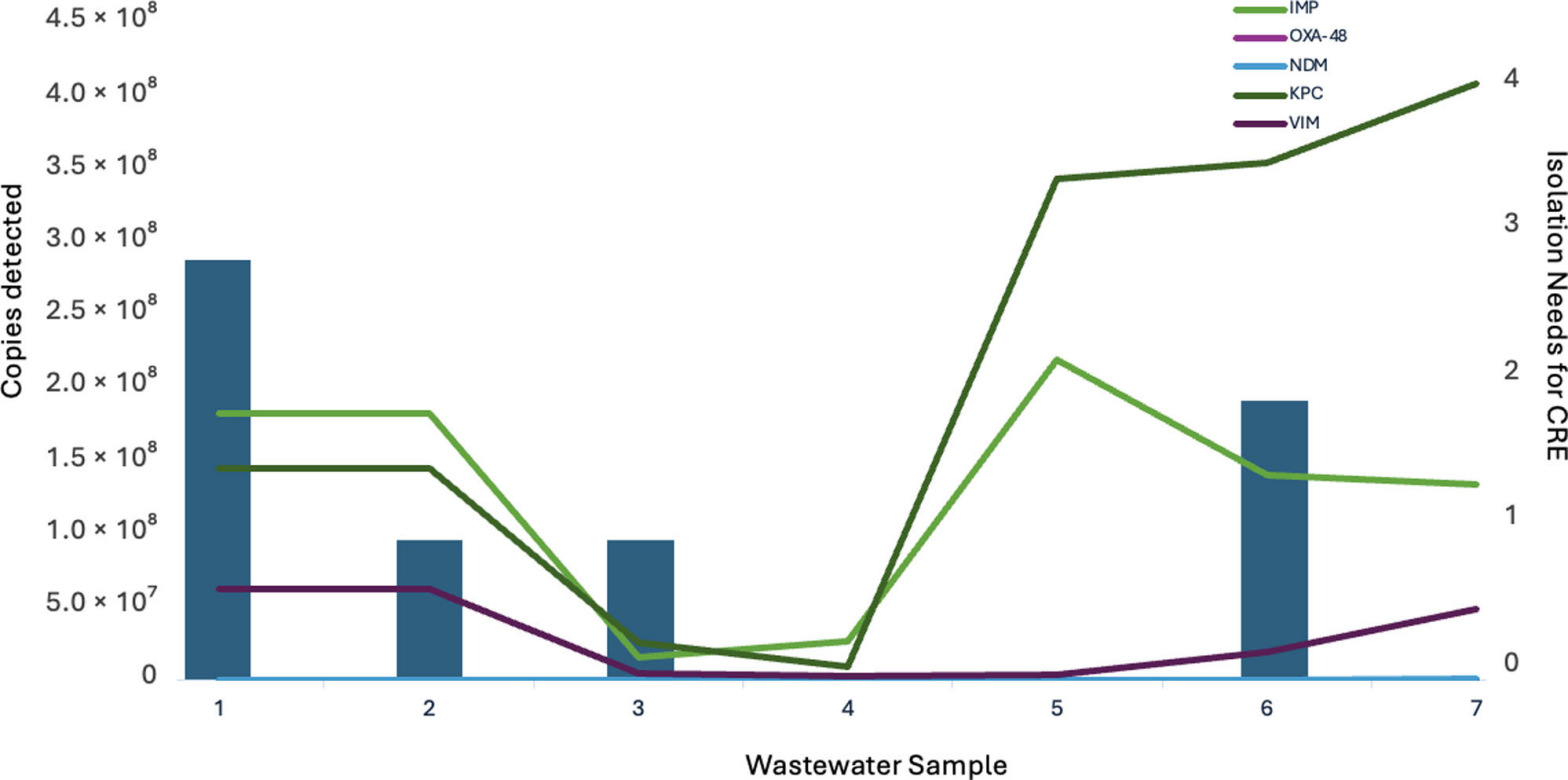

**Figure:**
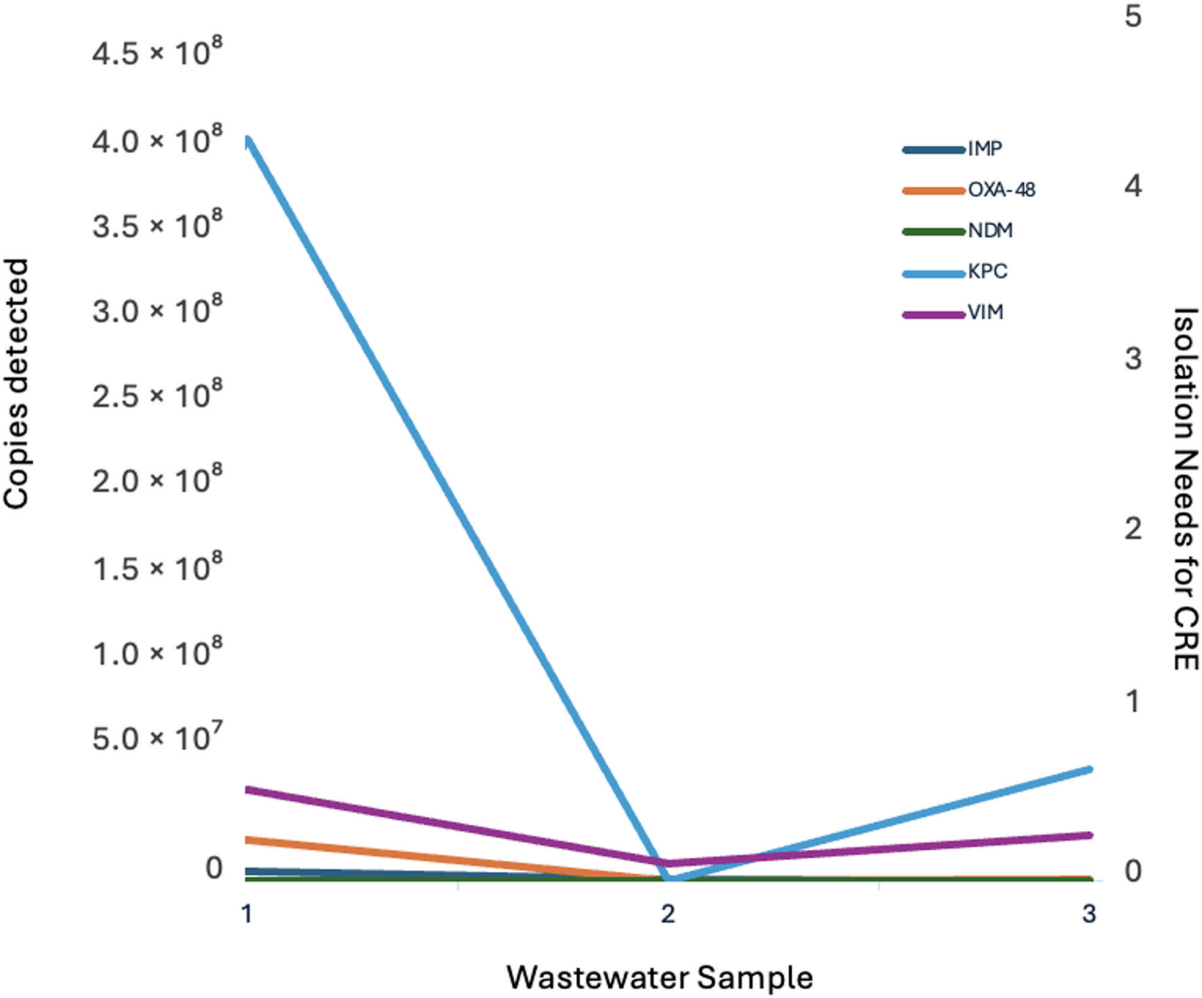

**Figure:**